# Boresight Calibration of Construction Misalignments for 3D Scanners Built with a 2D Laser Rangefinder Rotating on Its Optical Center

**DOI:** 10.3390/s141120025

**Published:** 2014-10-24

**Authors:** Jesús Morales, Jorge L. Martínez, Anthony Mandow, Antonio J. Reina, Alejandro Pequeño-Boter, Alfonso García-Cerezo

**Affiliations:** 1 Departamento de Ingeniería de Sistemas y Automática, Universidad de Málaga, Andalucía Tech, 29071-Málaga, Spain; E-Mails: jlmartinez@uma.es (J.L.M.); amandow@uma.es (A.M.); ajreina@uma.es (A.J.R.); gcerezo@ctima.uma.es (A.G.-C.); 2 Ingeniería UNO, Calle Alcalde Garret y Souto, 38, 29006-Málaga, Spain; E-Mail: info@ingenieriauno.com

**Keywords:** range sensors, system calibration, 3D laser scanner, plane detection, quality control

## Abstract

Many applications, like mobile robotics, can profit from acquiring dense, wide-ranging and accurate 3D laser data. Off-the-shelf 2D scanners are commonly customized with an extra rotation as a low-cost, lightweight and low-power-demanding solution. Moreover, aligning the extra rotation axis with the optical center allows the 3D device to maintain the same minimum range as the 2D scanner and avoids offsets in computing Cartesian coordinates. The paper proposes a practical procedure to estimate construction misalignments based on a single scan taken from an arbitrary position in an unprepared environment that contains planar surfaces of unknown dimensions. Inherited measurement limitations from low-cost 2D devices prevent the estimation of very small translation misalignments, so the calibration problem reduces to obtaining boresight parameters. The distinctive approach with respect to previous plane-based intrinsic calibration techniques is the iterative maximization of both the flatness and the area of visible planes. Calibration results are presented for a case study. The method is currently being applied as the final stage in the production of a commercial 3D rangefinder.

## 1. Introduction

Many promising applications of mobile robotics rely on three-dimensional (3D) data. Examples include warehouse automation [1], construction machinery [2], intelligent vehicles [3], planetary navigation [4], natural terrain exploration [5] and search and rescue [6].

A 3D range sensor provides distances to the closest objects within its measurement limits. The most mature and reliable 3D rangefinders are 3D laser scanners [2,7]. However, due to the cost of commercial solutions, many robotics researchers build 3D scanners by adding a rotation to off-the-shelf 2D rangefinders [8–10].

Performance of custom 3D devices depends both on the characteristics of the 2D sensor and on the implementation of the extra degree of freedom. From a functional standpoint, it is desirable that the optical center of the scanner coincides with that of the 2D device [11–14], even if many designs do not consider this alignment for the sake of mechanical simplicity [8–10,15–17]. This alignment allows the 3D device to maintain the same minimum range as the 2D scanner. It also avoids the use of offsets between the rotation and optical centers to obtain Cartesian coordinates. These actuated 2D LiDAR sensors require calibration to produce reliable point clouds.

Calibration of a 3D laser scanner can serve to obtain both intrinsic and extrinsic parameters. Intrinsic parameters are those related with the acquisition process and involve issues that are both temporal (*i.e.*, measurement synchronization) and geometric [18]. The intrinsic geometric parameters depend on the internal operation of the 3D scanner [19]. For instance, multi-beam 3D laser devices require calibration of a separate set of intrinsic parameters for each beam [20–23]. Extrinsic calibration refers to the geometric problem of positioning the sensor with respect to the mobile robot [24,25] or with respect to another sensor, like an inertial measurement unit (IMU) [26,27] or a camera [28,29]. Extrinsic calibration of a 3D laser scanner assumes that its internal parameters have been previously calibrated.

Even if some calibration methods have explored maximization of overall point cloud quality from several scans [18,25,30], most approaches are based on capturing particular objects. Among the latter, using artificial targets requires engineered environments [22,24,31–33]. For on-site calibration of high-end sensors in unprepared environments, plane-based calibration can offer equivalent results [34] by optimizing the flatness of detected planes [20,23,35].

The novelty of the solution proposed in this paper with respect to previous plane-based intrinsic calibration techniques [20–23,31,32,34,35] is the iterative maximization of both the flatness and the area of detected planar patches. The paper presents a practical intrinsic calibration procedure for 3D scanners with a low-cost 2D laser rangefinder rotating on its optical center. Inherited measurement limitations from this kind of 2D device prevent the estimation of very small translation misalignments, so the calibration problem reduces to obtaining boresight (*i.e.*, orientation) parameters. To this end, optimal parameters are obtained from a single 3D scan that contains at least one planar surface of unknown dimensions taken from an arbitrary position. The method is currently being applied as the final stage in the production of a commercial 3D rangefinder to control its quality.

The paper is organized as follows. The next section details the calibration procedure. Section 3 describes a case study for two units of the same 3D laser scanner model. The paper ends with conclusions, acknowledgments and references.

## 2. Calibrating Custom 3D Laser Scanners

### 2.1. Problem Statement

Commercial 2D devices are built with a rotating mirror, whose point of rotation is considered as the optical center *O*_2_ of the 2D device. Let the *Z*_2_ axis of the frame associated with the 2D device be coincident with the mirror rotation axis and the *Y*_2_ axis be aligned with the centerline of the measurement plane. Then, a point in the plane is given by its polar coordinates: angle *θ*, which is assumed to be null in the *X*_2_ direction, and range *ρ*.

**Figure 1.**
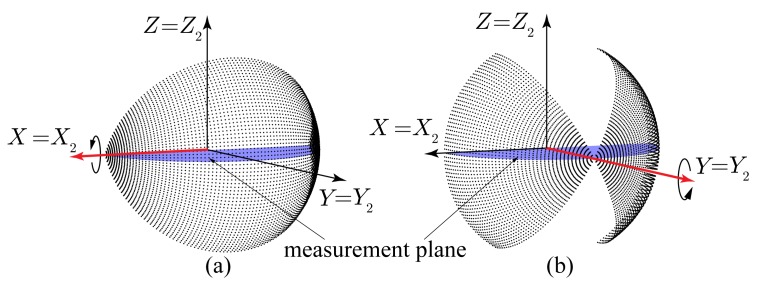
3D scanning configurations: (**a**) pitching; (**b**) rolling.

Two basic configurations are possible when using a 2D device to build a 3D scanner with the same optical center: pitching, by adding a rotation *β* around the *X*_2_ axis, and rolling, where rotation is introduced around the *Y*_2_ axis (see Figure 1). Revolution about the *Z*_2_ axis (*i.e.*, yawing) is not considered, as it is redundant with the 2D scanning plane. The proposed calibration procedure will be developed in the paper for pitching scanners, although it can be applied for both configurations.

In an ideal 3D sensor, its reference frame *OXY Z* is defined as coincident with that of the 2D device when *β* = 0°. This means that *X* and *X*_2_ should be perfectly lined up during pitching rotation, as shown in Figure 1a. Then, the Cartesian coordinates of the point cloud can be computed from *ρ, θ* and *β* as:

(1) $$\begin{array}{ccc} \left(\begin{array}{c} x\\ y\\ z \end{array}\right) & =\left(\begin{array}{cc} 1 & 0\\ 0 & C(\beta )\\ 0 & S(\beta ) \end{array}\right) & \left(\begin{array}{c} \rho C(\theta )\\ \rho S(\theta ) \end{array}\right) \end{array}$$

where *C*(·) and *S*(·) stand for cosine and sine functions, respectively. However, since the attachment of the 2D device to the rotating mechanism is not ideal in real sensors, *X*_2_ is not perfectly aligned with *X*. This misalignment provokes a distortion in the point cloud computed with Equation (1).

**Figure 2.**
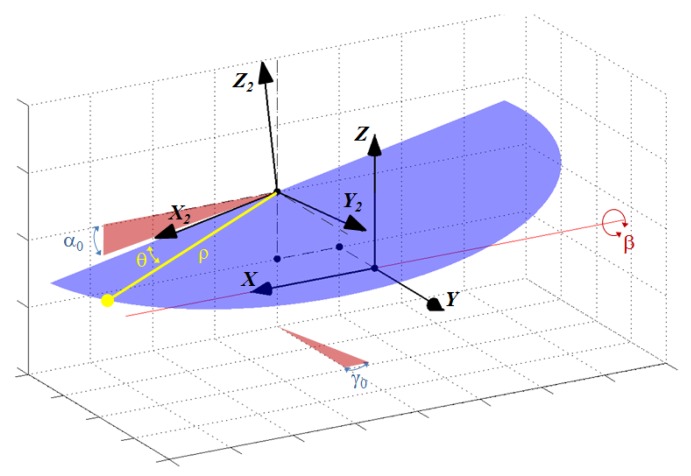
Misalignments between the 2D sensor frame and the 3D sensor frame.

Therefore, calibrating for misalignments is required to assess sensor construction quality and to produce a reliable point cloud. Calibration would imply computing the translation (*x*_0_, *y*_0_, *z*_0_) from *O*_2_ to *O*, as well as the rotation between frames. This rotation can be defined as a sequence of three intrinsic rotations *X-Y-Z* with angles *β*_0_, *α*_0_ and *γ*_0_, respectively (see Figure 2). Thus, in theory, a set of six parameters should be found.

### 2.2. Practical Considerations

Common off-the-shelf 2D scanners are affected by relevant range biases that depend not only on the distance to the target, but also to surface properties (e.g., color, material or brightness) and incidence angles. This bias is in the order of centimeters for Sick [36] and Hokuyo sensors [37,38]. Therefore, since translation misalignments (*x*_0_, *y*_0_, *z*_0_) are expected to be around a few millimeters, they cannot be estimated by using readings from the sensor itself in an unprepared environment, and the problem reduces to boresight calibration.

Regarding calibration parameters, *β*_0_ is special in that it does not provoke distortion, as it refers to the zero angle of the rotation mechanism. This parameter can be considered as part of the extrinsic calibration of the 3D sensor, *i.e.*, the relative transformation between the 3D sensor and the reference frame of the vehicle or the site where it is attached.

Taking into account these practical considerations, the calibration process can be actually simplified to obtaining only two intrinsic angles: *α*_0_ and *γ*_0_ (see Figure 2). After calibration, the following formula can be employed to obtain 3D Cartesian coordinates of a point in the 3D frame:

(2) $$\begin{array}{ccc} \left(\begin{array}{c} x\\ y\\ z \end{array}\right) & =\left(\begin{array}{cc} C(\alpha_{0})C(\gamma_{0}) & -C(\alpha_{0})S(\gamma_{0})\\ C(\Theta )S(\gamma_{0})+C(\gamma_{0})S(\alpha_{0})S(\Theta ) & C(\Theta )C(\gamma_{0})-S(\alpha_{0})S(\Theta )S(\gamma_{0})\\ S(\Theta )S(\gamma_{0})-C(\Theta )C(\gamma_{0})S(\alpha_{0}) & C(\gamma_{0})S(\Theta )+C(\Theta )S(\alpha_{0})S(\gamma_{0}) \end{array}\right) & \left(\begin{array}{c} \rho C(\theta )\\ \rho S(\theta ) \end{array}\right) \end{array}$$

where Θ = *β*_0_ + *β*.

### 2.3. Boresight Calibration Procedure

**Figure 3.**
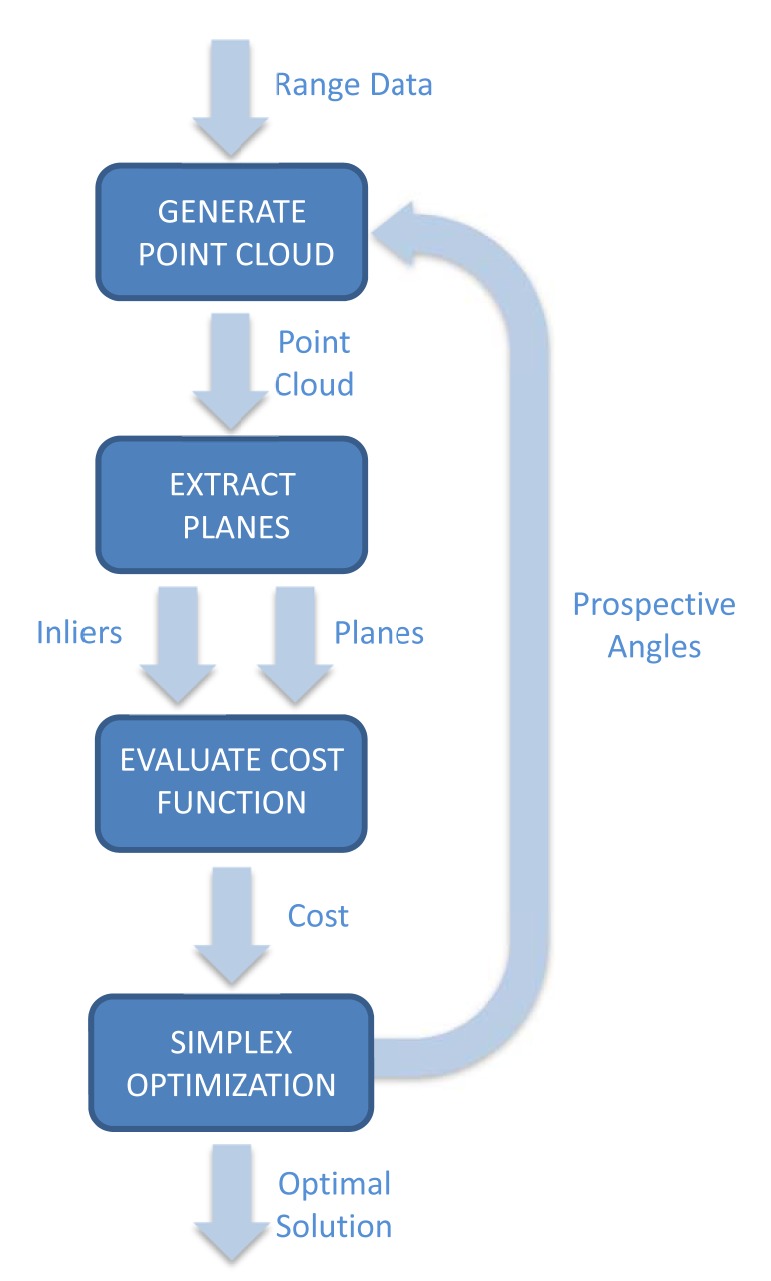
Outline of the calibration procedure.

The principle of the proposed calibration procedure is maximizing both the flatness and the area of detected planar surfaces in a single 3D scan. In particular, the well-known Nelder-Mead method [39] is adopted for this non-linear optimization process. The outline of the calibration procedure is sketched in Figure 3.

**Figure 4.**
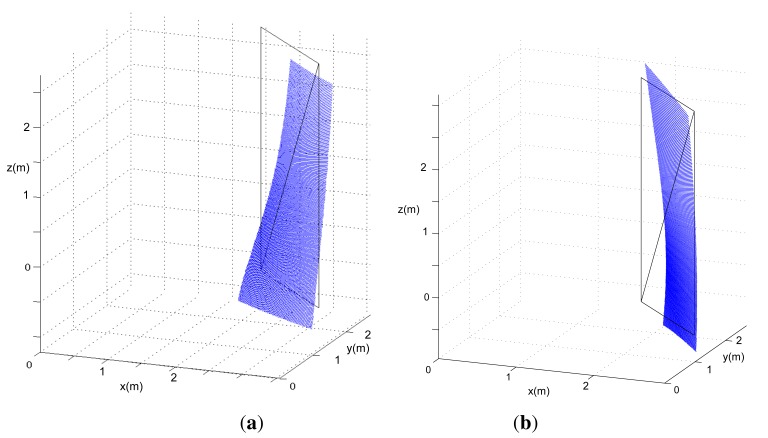
Simulated 3D scan of a planar surface scanned from a 3D device with (**a**) *α*_0_ = 10° and (**b**) *γ*_0_ = 10°. Null coordinates correspond to the origin of the optical frame of the 3D rangefinder.

The input to the procedure is the set of range data (*ρ, θ, β*) from a single 3D scan of an environment that contains planar surfaces, as commonly found in buildings. This scan does not need a prepared or ground truth environment. The only requirement is that there are planar surfaces in the sensor's field of view. Regarding the number and relative position of the planes, a simulation study has shown that just one surface suffices as long as its area is wide enough to evidence warp, but the use of more planes can enhance the calibration results. For instance, Figure 4 presents two simulation examples of the deformations experienced by the same plane surface when either *α*_0_ or *γ*_0_ are not null. As the effects of each parameter are different on the surface, simultaneous calibration of both angles is possible. Furthermore, there is no need to place the laser scanner in any particular pose with respect to the planar surfaces or to know their pose, material or dimensions.

The proposed algorithm consists of an iteration governed by the simplex method, which proposes prospective solutions. Given that there are two optimization parameters {*α*_0_, *γ*_0_}, the simplex is composed of three vertices, which are initialized randomly around zero values. Then, each iteration processes one vertex through four major steps.

The first step is computing the Cartesian coordinates with Equation (2) from range data according to prospective values of {*α*_0_,* γ*_0_}. The angle *β*_0_ is set to a constant value (e.g., zero, for simplicity).

In the second step, segmentation of the point cloud is performed to extract planes using the random sample consensus (RANSAC) method [40] implemented in the Point Cloud Library [41]. The output of the RANSAC function for plane detection only contains inliers of detected planes and their corresponding equations. When the plane is distorted due to erroneous calibration parameters, the size of the planar patches defined by their corresponding inliers can be small. The user must indicate the number of planar surfaces *P* to be extracted by this segmentation algorithm and the distance threshold *τ* for inliers. Then, RANSAC returns the *P* planes with a greater number of inliers.

The third step is the evaluation of the cost function *E* to be minimized. This function is defined as:

(3) $$E=N\sum_{j=1}^{P}\left(\frac{1}{N_{j}^{2}}\sum_{i=1}^{N_{j}}d_{j,i}\right)$$

where *N* is the total number of valid ranges (*i.e.*, after discarding erroneous and out-of-range readings, such as the sky), *N_j_* is the number of inliers within the *j*-th planar surface and *d_j,i_* is the distance of the *i*-th inlier to its corresponding (*j*-th) planar surface. Function *E* consists of the sum of distances of the inliers to their respective planes divided by the square of the number of inliers. In this way, apart from reducing the mean error between points and planes, the number of inliers, which is an indication of the total area of planar patches, is also maximized.

Finally, the Nelder–Mead method proposes new vertices to replace the worst valued vertices of the simplex until either all vertices are closer than a given threshold or a maximum number of iterations is reached.

## 3. Case Study

### 3.1. 3D Laser Scanner

**Figure 5.**
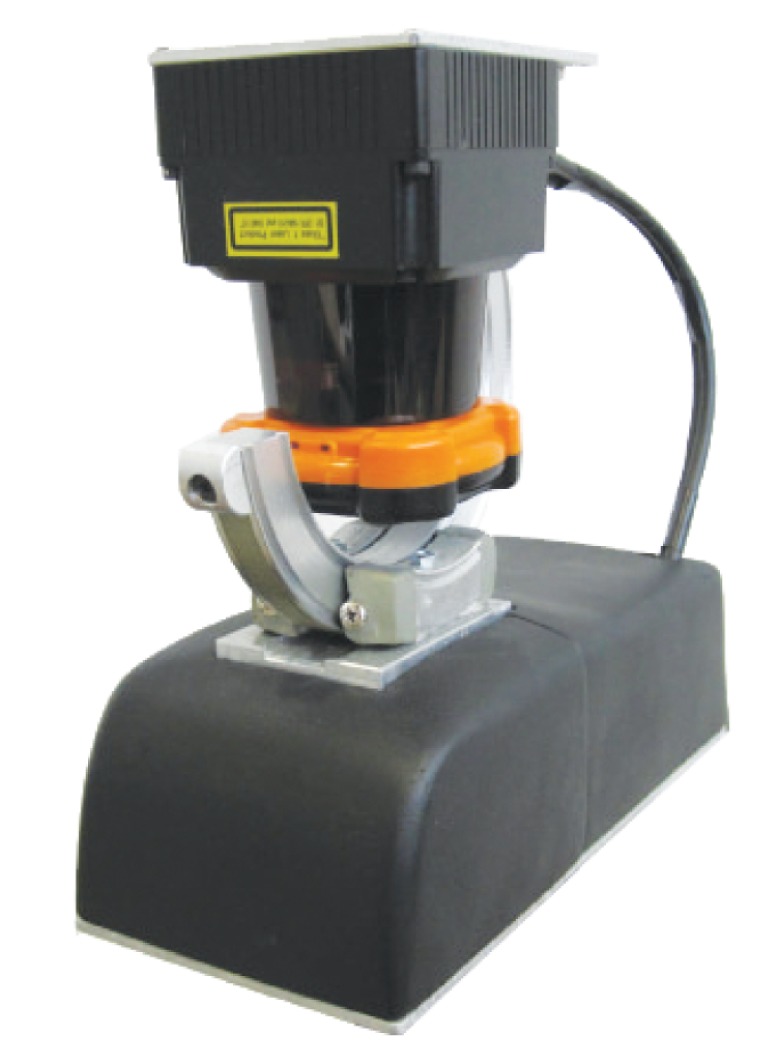
The UNOlaser rangefinder.

**Figure 6.**
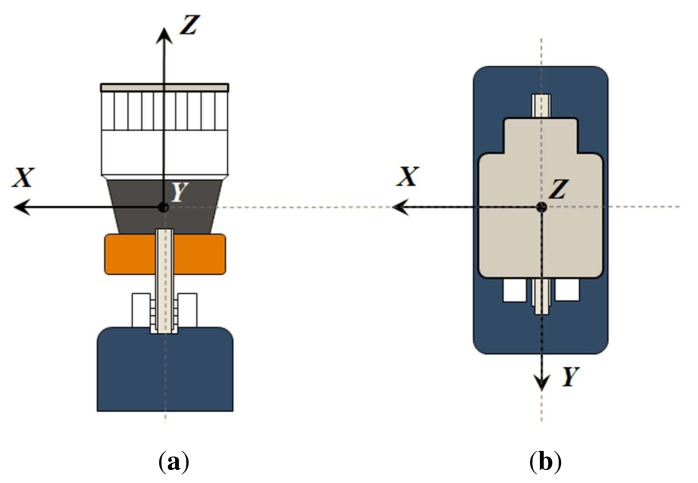
UNOlaser reference frame: front (a) and top (b) views.

The custom-made 3D laser rangefinder used in the case study is commercially available under the product name UNOlaser (see Figure 5). This sensor is based on pitching the Hokuyo UTM-30LX laser rangefinder around its optical center [42]. The reference frame *OXY Z* for the 3D sensor (see Figure 6) has been defined as explained in Section 2. The device has been already employed for the classification of terrain elevations [5], to register 3D point clouds [43] and to analyze the navigability of natural terrain [44].

The Hokuyo 2D rangefinder has compact dimensions (30 × 60 × 87 mm) and a light weight (370 g). 2D scans are produced in 25 ms with a field of view of 270°, an angular resolution of 0.25° and maximum and minimum scanning ranges of 30 m and 0.1 m, respectively. This sensor is suitable for scanning both indoor and outdoor environments [44].

The 3D laser rangefinder has been designed to get the most of the Hokuyo sensor performance; especially its large 2D field of view and its fast response. Nevertheless, the 3D sensor inherits the measurement characteristics of the Hokuyo UTM-30LX, whose ranges are subject to biases of ±3 cm that depend on target properties, distance and incidence angles [38]. Under the same measurement conditions, ranges approximately follow a Gaussian distribution around their corresponding biases.

The 3D device weighs 850 g, and its maximum dimensions are 182 × 80 × 191 mm. It is powered by a DC supply of 12 V with a nominal consumption of 14.4 W with peaks of 33.6 W. The maximum sweep of pitch angles is 129°. A complete 3D scan can be obtained with a maximum pitch resolution of 0.067367° in 95.75 s and with a minimum pitch resolution of 4.16129° in 1.55 s.

### 3.2. Calibration Results

**Figure 7.**
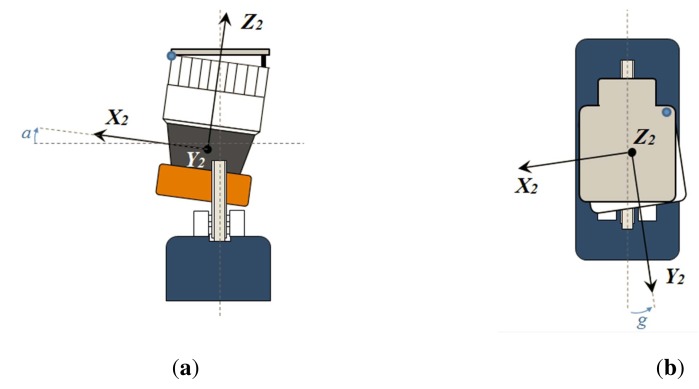
Two types of mechanical misalignments intentionally introduced in UNOlaser: angles *a* (**a**) and *g* (**b**)

Two units of the UNOlaser have been employed in the experiments. Apart from calibrating both sensors as delivered, two kinds of misalignments have been intentionally introduced into the attachment of the 2D sensor. Concretely, discrepancies are set up by partially unscrewing the 2D scanner to a plate of the extra rotation mechanism (see Figure 7). The resulting misalignments are the angles *a* and *g*, which contribute to *α*_0_ and *γ*_0_ errors, respectively. Furthermore, two independent calibrations have been performed for each combination. Besides, two different values of *P* have been considered. All in all, the case study has considered 72 calibration experiments.

**Figure 8.**
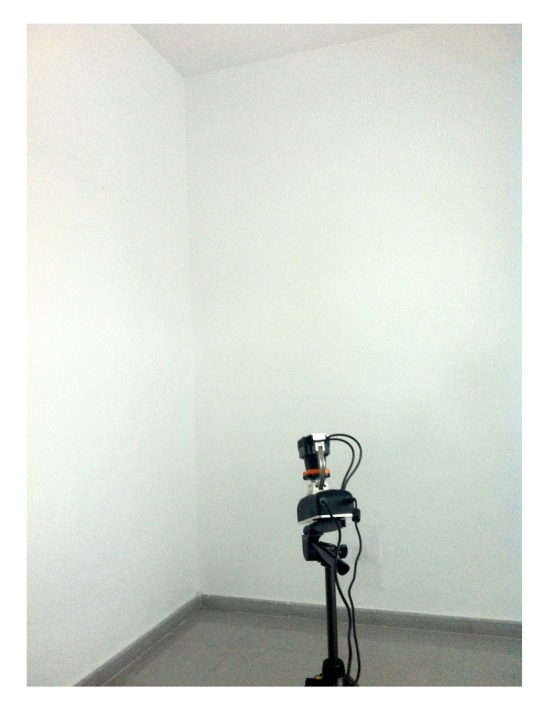
The corner of the office where 3D scans were taken.

For each calibration, a single scan with visible planar surfaces has been obtained from an office corner, as shown in Figure 8. All of the scans where taken with a vertical resolution of 0.274°, which is similar to the 2D horizontal resolution. In this scene, at least four planar surfaces are visible (*i.e.*, two walls, the floor and the ceiling), so *P* = 4 is a reasonable value for the calibration procedure. In addition, the performance of the method has been tested for the less favorable case of considering only one plane (*P* = 1). The inlier discrimination threshold has been set to *τ* = 1 cm.

Tables 1 and 2 show the calibrations results for both units. The first row in the tables, *i.e., a* = *g* = 0°, refers to the calibration of the 3D devices as delivered. In these cases, the calibration indicates small errors under one degree. Interestingly, calibration also reveals similar misalignments for both units, which can be attributed to repeatability in the construction procedure. Besides, intentional errors are correctly detected by the calibration procedure. Moreover, the two different calibrations for each misalignment configuration produce similar results. Regarding the number of planar surfaces, in general, both *P* = 1 and *P* = 4 produce similar results, with some improvements in the latter.

**Figure 9.**
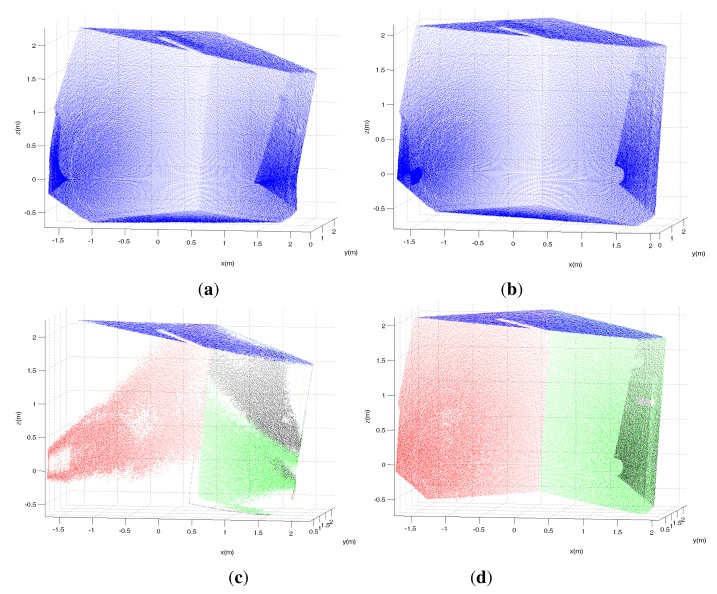
The point cloud obtained by the first unit with a *a* = 0°, *g* = 5.78° misalignment before (**a**) and after (**b**) calibration with *P* = 4. The inliers in detected planar patches are shown in red, blue, green and black before (**c**) and after (**d**) calibration.

Figure 9 illustrates a calibration example with a large misalignment between the 2D and 3D rotation axes. The warp in planar surfaces, which is evident in Figure 9a, is corrected when applying the optimized calibration parameters in Figure 9b. The principle of the proposed method is illustrated in Figure 9c and d. The four planar patches returned by RANSAC for the uncalibrated scan are depicted in Figure 9c with different colors. Note that only inlier points are shown in this figure and that, due to warp, the wall on the right is considered as two different planar patches. At the end of the calibration process (see Figure 9d), a single planar patch (represented in green) corresponds to the whole area of this wall. Furthermore, the four planar patches from the calibrated point cloud correspond to the areas of the four planar surfaces with a greater number of scanned points.

### 3.3. Verification

**Figure 10.**
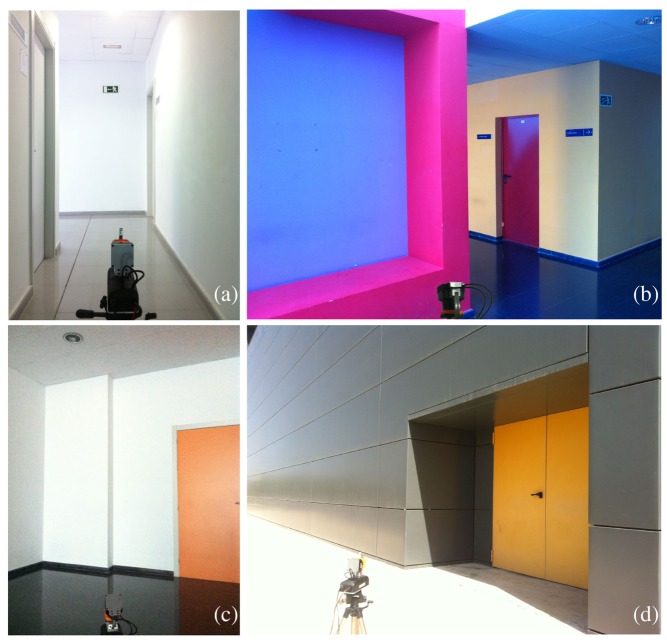
Verification experiment scenes: corridor (**a**); hall (**b**); room (**c**) and outdoor building front (**d**).

Scans taken in different environments than the one used for calibration have served to verify calibration results for both units as delivered. In particular, the calibration parameters are those in the first row of Tables 1 and 2 for the first scan and *P* = 4; *i.e*., *α*_0_ = 0.28° and *γ*_0_ = 0.56° for the first unit and *α*_0_ = 0.39° and *γ*_0_ = 0.64° for the second. Cost function *E* has been obtained with Equation (3), *τ* = 1 cm and *P* = 4 from point clouds computed with both Equations (1) and (2) for indoor and outdoor scans (see Figure 10). The results are given in Table 3. This table also compares the total rate *R* of inliers in the *P* planes with respect to *N*:

(4) $$R=\frac{\sum_{j=1}^{P}N_{j}}{N}\times 100$$

and the standard deviation *σ* of the distance of inliers to their respective planes:

(5) $$\sigma =\sqrt{\frac{\sum_{j=1}^{P}\sum_{i=1}^{N_{j}}d_{j,i}^{2}}{\sum_{j=1}^{P}N_{j}}}$$

In all of the validation environments, *E* is improved when calibration parameters are employed, which means that warp has been reduced in the detected planes. Furthermore, the increase of the rate of inliers *R* and the general decrease of *σ* corroborate the warp reduction.

## 4. Conclusions

Off-the-shelf 2D scanners customized with an extra rotation are commonly employed to obtain 3D range data in many research applications. However, construction misalignments in the attachment of the 2D device to the rotation mechanism provoke distortions in the point cloud. Therefore, calibrating for misalignments is important, both to assess sensor construction quality and to improve the reliability of point clouds.

The paper has proposed a simple intrinsic calibration procedure to compute construction misalignments for 3D sensors where the extra rotation is aligned with the optical center. Inherited measurement limitations from the 2D device prevent the estimation of very small translation misalignments, and the calibration problem reduces to obtaining boresight parameters. The method is based on detecting plane surfaces from a single scan and optimizing calibration angles to maximize both the number of inliers and the flatness. The calibration scan can be taken from an arbitrary position in an unprepared environment as long as at least one planar surface is visible. Thus, the method can be practically applied without the need of additional equipment in urban environments. Successful calibration results are presented for a commercial 3D rangefinder with the pitching configuration.

The proposed method is currently being applied as the final stage in the production of this scanner to verify the lack of construction failures. Future work includes the application of the proposed method to calibrate a new Hokuyo-based 3D rangefinder with the rolling configuration.

## Figures and Tables

**Table 1.** Calibrated parameters with and without misalignments for the first unit of UNO-laser.

		*P* = 1						*P* = 4					
Misalignments		First Scan			Second Scan			First Scan			Second Scan		
*a*(°)	*g*(°)	*α*_0_(°)	*γ*_0_(°)	*E*·10^3^	*α*_0_(°)	*γ*_0_(°)	*E*·10^3^	*α*_0_(°)	*γ*_0_(°)	*E*·10^3^	*α*_0_(°)	*γ*_0_(°)	*E*·10^3^
0	0	0.53	0.44	10.1	0.52	0.33	10.1	0.28	0.56	4.49	0.33	0.58	4.50
1.71	0	2.14	0.46	10.2	2.21	0.47	10.3	1.95	0.29	4.48	2.03	0.39	4.51
3.91	0	4.05	0.28	10.3	4.06	0.36	10.1	3.88	0.28	4.49	3.88	0.28	4.49
−1.69	0	−1.19	0.39	10.2	−1.18	0.38	10.2	−1.37	0.39	4.55	−1.15	0.35	4.54
−3.92	0	−3.20	0.29	10.2	−3.21	0.27	10.2	−3.30	0.15	4.58	−3.20	0.37	4.61
0	1.84	0.46	2.36	10.6	0.46	2.39	10.5	0.34	2.38	4.51	0.29	2.23	4.53
0	5.74	0.51	6.02	10.3	0.46	6.01	10.3	0.23	5.95	4.53	0.39	5.93	4.47
0	−1.88	0.38	−1.82	10.4	0.40	−1.89	10.4	0.41	−1.97	4.52	0.34	−1.98	4.51
0	−5.48	0.38	−5.19	10.5	0.32	−5.21	10.7	0.30	−5.33	4.55	0.41	−5.30	4.55

**Table 2.** Calibrated parameters with and without misalignments for the second unit of UNO-laser.

		*P* = 1						*P* = 4					
Misalignments		First Scan			Second Scan			First Scan			Second Scan		
*a*(°)	*g*(°)	*α*_0_(°)	*γ*_0_(°)	*E*·10^3^	*α*_0_(°)	*γ*_0_(°)	*E*·10^3^	*α*_0_(°)	*γ*_0_(°)	*E*·10^3^	*α*_0_(°)	*γ*_0_(°)	*E*·10^3^
0	0	0.47	0.86	9.11	0.51	0.83	9.12	0.39	0.64	4.34	0.33	0.51	4.35
1.97	0	2.21	0.96	9.17	2.19	1.04	9.20	2.16	0.77	4.36	2.08	0.85	4.40
3.66	0	4.05	0.9	10.0	4.07	0.95	10.2	3.93	0.71	4.52	3.84	0.60	4.57
−1.82	0	−1.39	0.77	9.38	−1.44	0.83	9.36	−1.50	0.57	4.43	−1.55	0.65	4.44
−3.7	0	−3.23	0.95	9.98	−3.26	0.90	9.93	−3.21	0.55	4.56	−3.34	0.69	4.54
0	2.03	0.53	2.97	9.25	0.47	2.94	9.44	0.52	2.65	4.43	0.44	2.84	4.46
0	5.33	0.49	6.18	9.35	0.51	6.14	9.33	0.51	5.97	4.44	0.45	6.00	4.44
0	−1.9	0.43	−0.99	9.43	0.41	−1.07	9.50	0.20	−1.46	4.46	0.28	−1.19	4.46
0	−5.48	0.40	−4.63	9.39	0.31	−4.58	9.46	0.30	−4.77	4.44	0.28	−4.84	4.45

**Table 3.** Verification results.

		Uncalibrated			Calibrated		
Laser Scanner	Scene	*E*·10^3^ Equation (1)	*R*(%) Equation (4)	σ(mm) Equation (5)	*E*·10^3^ Equation (2)	*R*(%) Equation (4)	σ(mm) Equation (5)
First Unit	Corridor	5.21	80.00	4.50	3.98	82.11	4.04

	Hall	6.57	69.40	4.69	5.01	73.93	4.51

	Room	5.87	76.90	4.84	4.47	83.26	4.53

	Outdoor	8.42	62.86	5.30	6.90	66.08	5.36

Second Unit	Corridor	5.81	74.51	4.58	4.72	76.49	4.40

	Hall	6.64	65.80	4.55	4.95	74.08	4.47

	Room	6.38	74.19	4.94	4.55	82.92	4.59

	Outdoor	8.95	59.50	5.48	7.05	61.67	5.16
